# Caroli’s Syndrome: An Early Presentation

**DOI:** 10.7759/cureus.11029

**Published:** 2020-10-18

**Authors:** Elsa Acevedo, Stephanie S Laínez, Pablo Andrés Cáceres Cano, Daniel Vivar

**Affiliations:** 1 General Medicine, National Autonomous University of Honduras, Tegucigalpa, HND; 2 Gastroenterology, María Hospital of Pediatric Specialties, Tegucigalpa, HND

**Keywords:** caroli’s syndrome, congenital hepatic fibrosis, autosomal recessive polycystic kidney, fibropolycystic liver disorders

## Abstract

Fibropolycystic liver disorders (FLD) arise from abnormal development of the ductal plate and are classified according to the size of the affected hepatobiliary duct. Congenital hepatic fibrosis (CHF) has small duct involvement characterized by a variable degree of periportal fibrosis and hyperplasia without affecting the liver’s architecture. Caroli’s disease (CD) is a rare autosomal recessive disorder with a prevalence of one case per 1,000,000 people and is characterized by cystic dilation of large intrahepatic ducts. When the disease presents with congenital hepatic fibrosis, it is referred to as Caroli’s syndrome (CS). Patients are usually diagnosed around the age of 20 with episodes of cholangitis, portal hypertension or hepatomegaly. We present the case of a two-year-old male with a previous history of autosomal recessive polycystic kidney disease (ARPKD) who presented to the emergency room with variceal bleeding secondary to portal hypertension. The physical examination showed an acutely ill-looking boy, with evident paleness and distended abdomen. Past medical history was negative for previous gastrointestinal bleeding or episodes of cholangitis. An upper gastrointestinal endoscopy was performed, showing esophageal varices secondary to portal hypertension. Imaging studies revealed hepatosplenomegaly, alterations in liver echogenicity, and dilated saccular bile ducts affecting both liver lobes without observing any apparent obstruction, highly suggestive of CD. A liver biopsy revealed nodular liver tissue with marked fibrosis between nodules, which confirmed the presence of CHF. Both kidneys were increased in size, hyperechoic and with loss of corticomedullary differentiation. FLD commonly present with coexisting hepatobiliary and renal alterations. Therefore, starting at the time of initial diagnosis, all patients with ARPKD should be evaluated to detect liver abnormalities due to the high association. Despite the rarity of CS, especially in early childhood, the association between ARPKD and FLD is well documented. So if this clinical presentation arises, CS should be suspected.

## Introduction

Fibropolycystic liver disorders (FLD) are a group of pathologies that result from abnormal embryological development of the ductal plate and lead to various degrees of duct pathology such as dilatation and cyst formation [1]. They are classified according to the size of the embryological hepatobiliary duct involved [1]. Conditions involving the smallest immature intrahepatic bile duct are called congenital hepatic fibrosis (CHF) [2]. The true incidence of CHF is unknown; however, it is believed to be one in 10,000 to 20,000 live births [2]. Caroli’s disease (CD) is a rare autosomal recessive disease with a prevalence of one in 1,000,000, characterized by cystic dilatation of large intrahepatic bile ducts [3]. Although the disease is present at birth, it is typically not clinically significant for the first two decades of life [4]. CD can present as a standalone or may co-exist with CHF, and is thus termed Caroli's syndrome [3]. They are genetically related to autosomal recessive polycystic kidney disease (ARPKD). CD and CHF are characterized by recurrent acute cholangitis or severe portal hypertension respectively [5]. We describe a unique case of CS diagnosed after a two-year-old male with ARPKD presented with upper gastrointestinal bleeding secondary to portal hypertension.

## Case presentation

A two-year-old male patient presented to the emergency room with a two-day history of melena and hematemesis. His prenatal, birth history and neonatal period were unremarkable. At one year of age, he was diagnosed with polycystic kidneys. However, the parents did not follow up with scheduled appointments. He had no history of previous episodes of abdominal pain or jaundice. The physical examination showed an acutely ill-looking boy, with a protuberant abdomen, non-icteric, asthenic, and with evident paleness. His weight and height were 11 kg and 82 cm, at 10th and 9th percentile for age respectively. His abdomen was distended with an abdominal circumference of 56 cm with hepatomegaly of 7 cm below the costal margin and splenomegaly of 4 cm below the costal margin.

Laboratory studies revealed a hemoglobin level of 9.9 g/dL, hematocrit of 26.8%, leukocyte count of 9460 /µL, creatinine of 1.1 mg/dL, blood urea nitrogen (BUN) of 74 mg/dL, alanine aminotransferase (ALT) of 32 UI/L, alkaline phosphatase of 196 UI/L. 

An upper gastrointestinal endoscopy showed esophageal varices secondary to portal hypertension (Japanese classification FIII) which were subsequently ligated. Serology for hepatitis B, hepatitis C, Epstein-Barr virus, and cytomegalovirus were negative. Other negative test results included thick blood smear, latex agglutination test, anti-Smith antibodies, hemoglobin electrophoresis, and liver and kidney type 1 anti-microsomal antibody (anti-LKM-1). His serum ferritin levels, transferrin saturation index, and ceruloplasmin were within normal limits.

Abdominal ultrasound revealed an enlarged liver (size 11.1 cm) with alteration in its echogenicity, defined borders, no masses or cysts, and a patent portal vein measuring 6.2 mm. The spleen was enlarged, measuring 9.4 x 3.7 cm, which appeared homogenous with no focal or diffuse lesions. The bile duct was not dilated. Both kidneys were increased in size, hyperechoic, and with loss of corticomedullary differentiation. There were no stones or dilation of the collecting system. A normal abdominal Doppler ultrasound and transthoracic echocardiography ruled out Budd Chiari syndrome.

**Figure 1:**
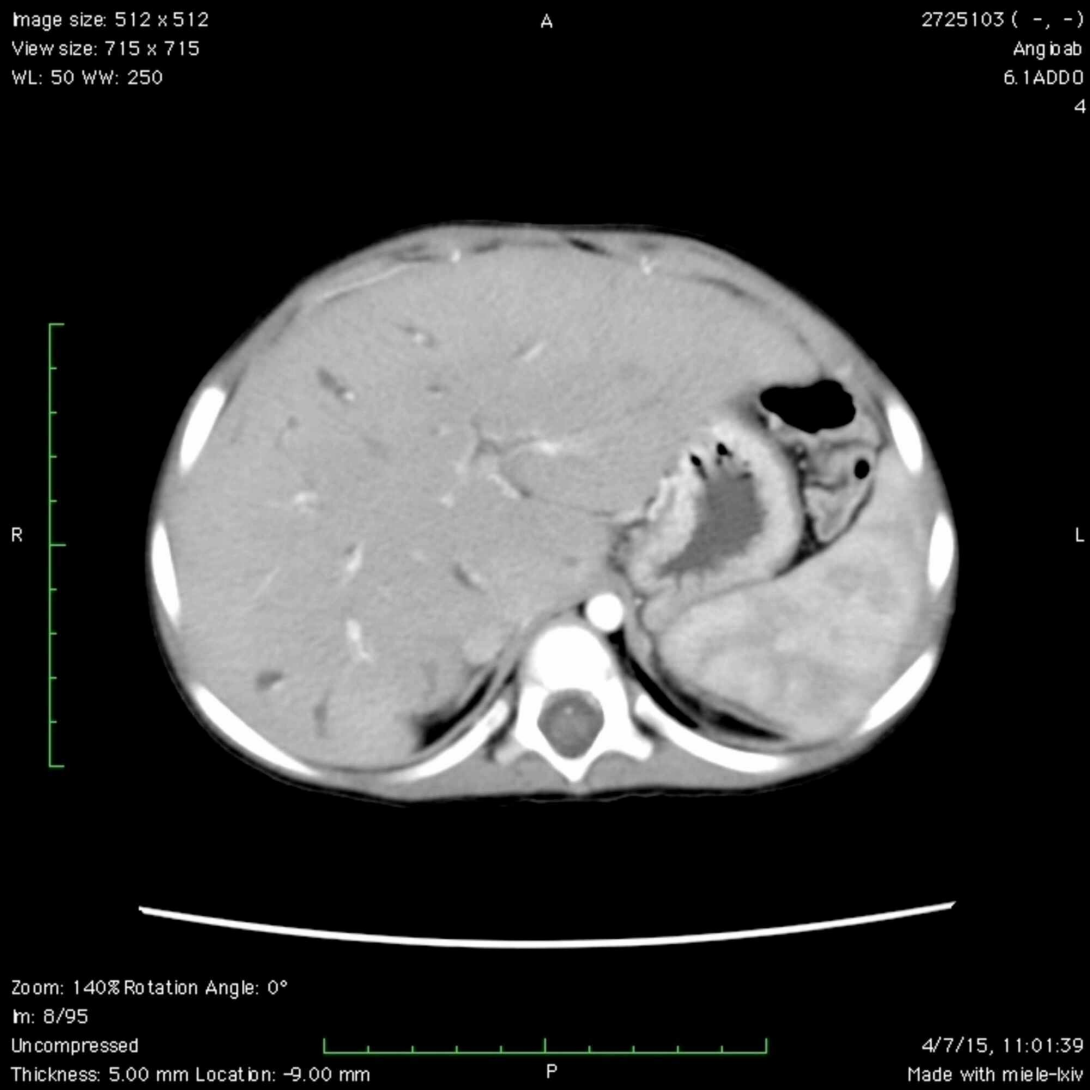
Computed tomography showing dilated saccular bile ducts

**Figure 2:**
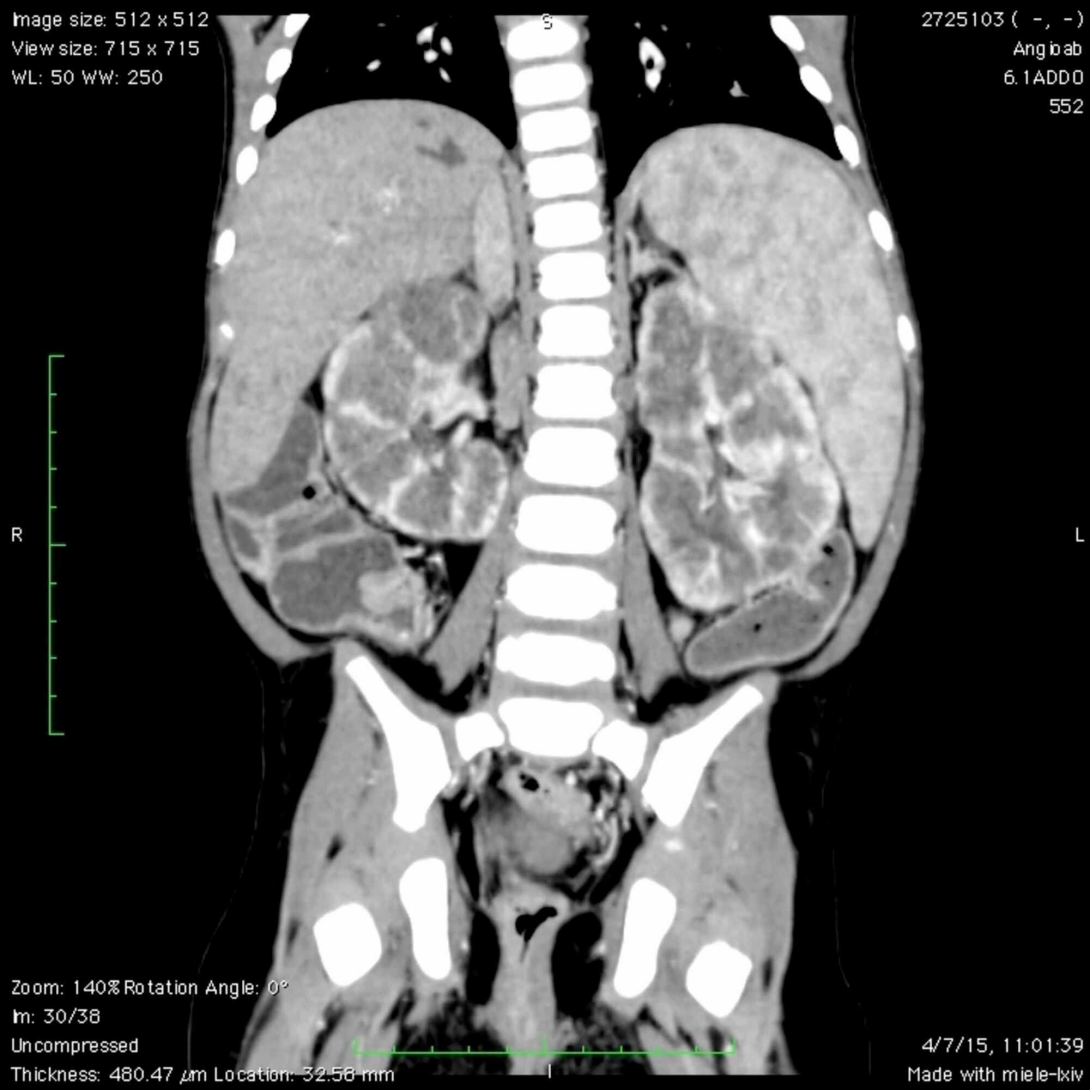
Computed tomography showing polycystic kidneys

Due to the inconclusive results of the abdominal ultrasound and Doppler, a computed tomography (CT) angiography and liver biopsy were requested. The CT angiography found significant hepatomegaly with a craniocaudal diameter at the level of the right hepatic lobe that reached 11.4 cm (normal 6-10 cm) and splenomegaly with a longitudinal diameter of 13.1 cm (normal 5-8 cm). Multiple intrahepatic images were found which corresponded to the dilated saccular bile ducts. Both liver lobes were affected, and there was no apparent obstruction. Dilatation of the portal vein was also observed, with a transverse diameter of up to 10 mm, and large esophageal varices. The common bile duct was dilated with a transverse diameter of 7 mm. Finally, important vascularization at the level of the hemorrhoidal plexus was observed. The concluding findings were highly suggestive of CD with evident signs of portal hypertension, vesicular hydrops, multiple mesenteric and retroperitoneal lymphadenopathy, and polycystic kidney disease (Figures 1, 2).

**Figure 3:**
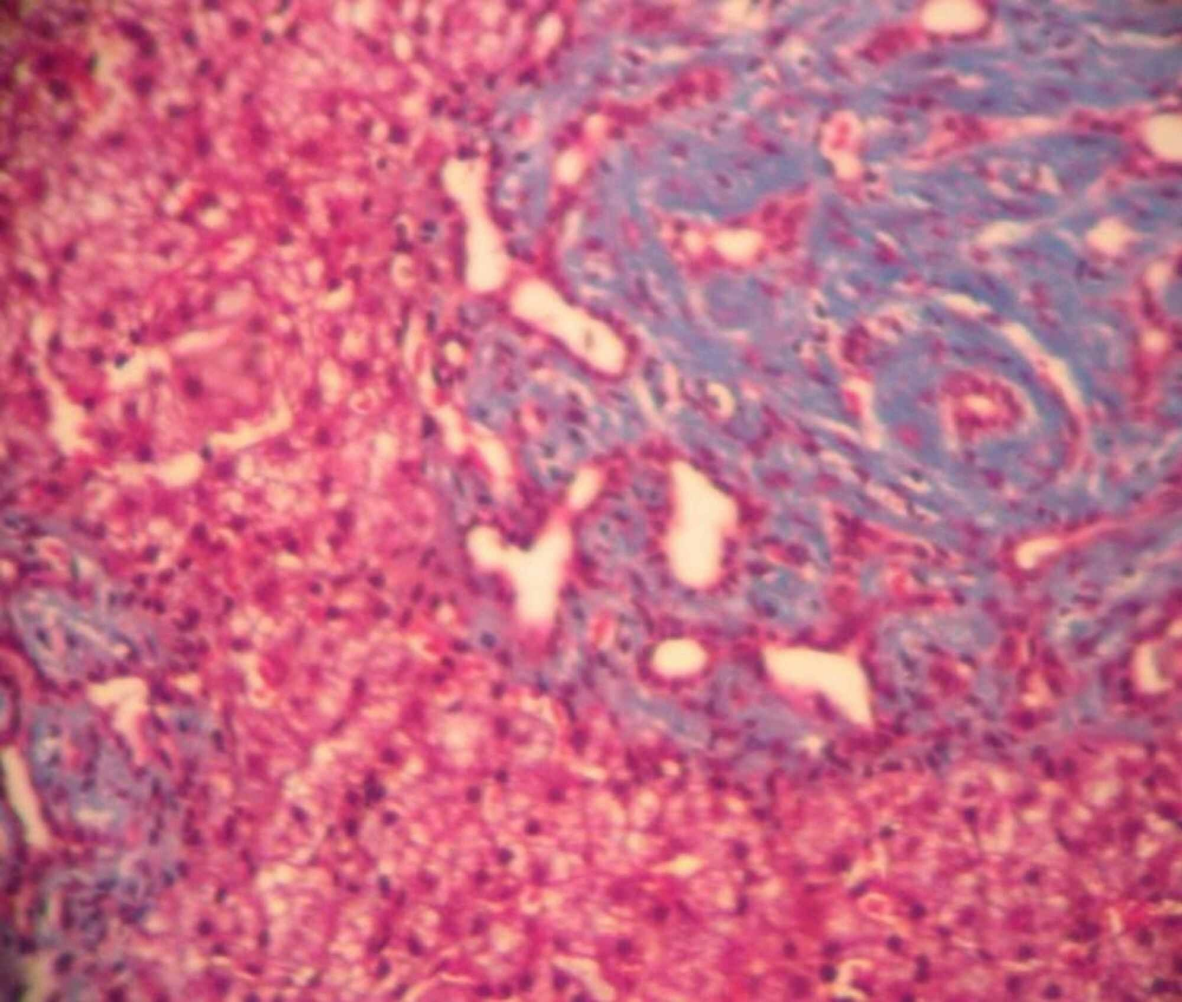
Liver biopsy The liver tissue is nodular in appearance with well-defined borders, without inflammation. There is marked fibrosis between the nodules, which is stained with Masson’s trichrome.

**Figure 4:**
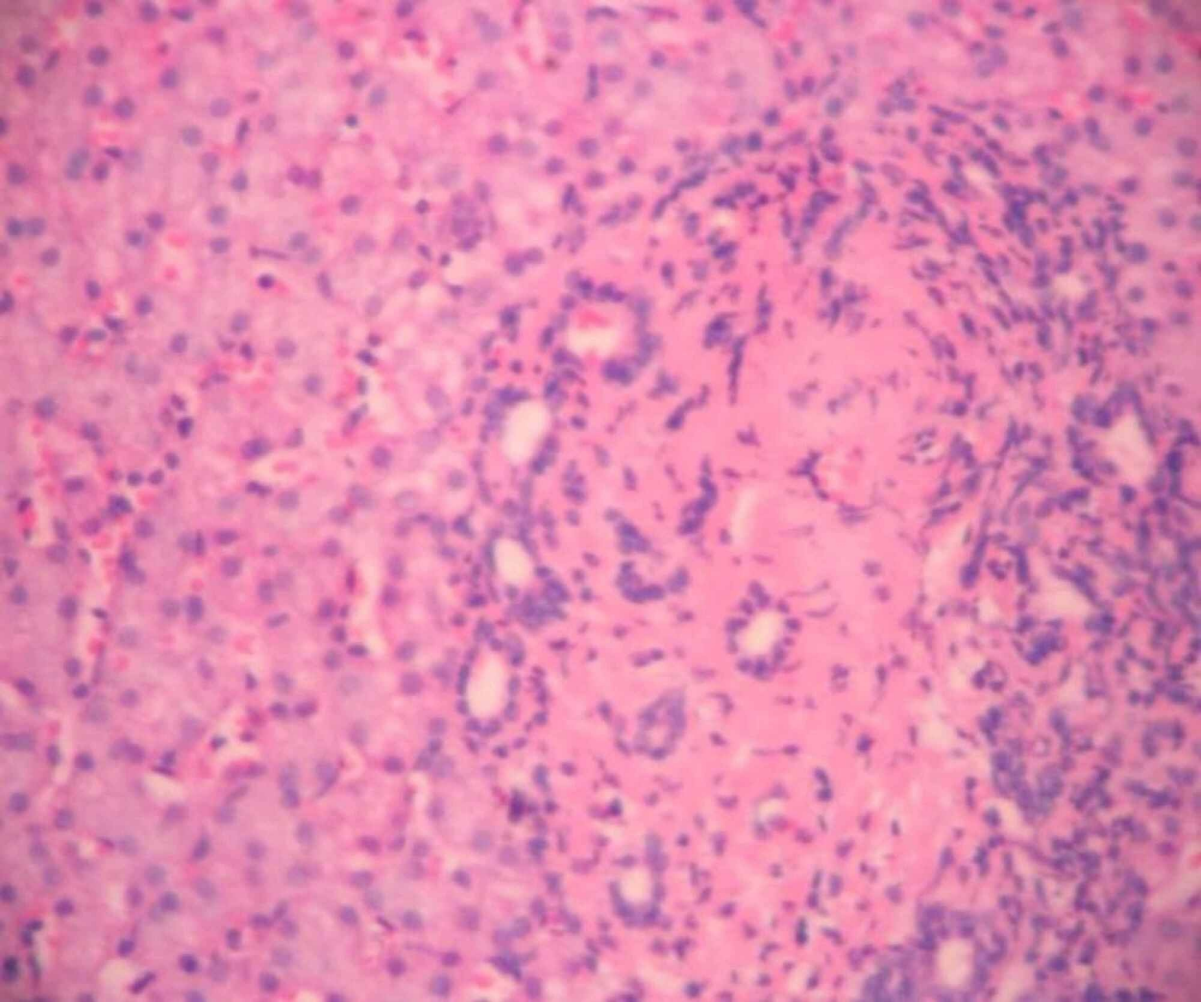
Liver biopsy Marked ductal proliferation and focal cholestasis (Hematoxylin-Eosin staining).

A liver biopsy revealed nodular liver tissue with marked fibrosis between nodules, which confirmed the presence of CHF (Figures 3, 4). The discharge treatment was symptomatic, including antihypertensive drugs, beta-blockers for portal hypertension, and a proton pump inhibitor. 

## Discussion

The biliary tree develops from a layer of cells called the ductal plate at nine weeks gestation, after which undergoes morphological remodeling and partial involution [2]. FLDs arise from malformations in the different stages of ductal plate remodeling [1]. These disorders tend to coexist with hepatobiliary and renal alterations. They are classified according to the size of the embryological hepatobiliary duct involved. Small duct involvement includes CHF and biliary hamartomas, while large duct involvement includes CD (intrahepatic ducts) and choledochal cyst (extrahepatic ducts) [1].

CHF is characterized by a variable degree of periportal fibrosis and hyperplasia of the bile ducts that does not alter the architecture of the liver. However, it alters the venous resistance of the portal branches leading to the development of portal hypertension [5,6]. CHF is often misdiagnosed as cirrhosis, despite the differentiating factor that hepatocellular function remains normal in CHF [5]. To our knowledge, there are two reported cases in the English literature of pure CHF presenting with hepatic failure and cirrhosis and a report of an Iranian woman who developed cirrhosis and subsequently hepatocellular carcinoma [7,8]. CHF is usually diagnosed during adolescence or young adulthood, and shows equal distribution between sexes [5,6]. Four clinical presentations are detailed in the literature and include portal hypertension, cholangitic, mixed form, and the latent-presentation at a late age [6]. The most common presentation is portal hypertension with consequent development of esophageal varices, which may result in hematemesis or melena [6,9]. The patient in our case became symptomatic at an unusual age but with a typical clinical presentation. Similar findings were seen in a retrospective case review study of children with CHF conducted in Birmingham Children’s Hospital, where portal hypertension resulting in hypersplenism, esophageal varices, and GI bleeding was documented in the majority of the patients [10]. Hepatomegaly (usually hypertrophic left segment and atrophic right lobe) with a firm to hard liver on palpation and nephromegaly in those having ARPKD are the additional physical findings in CHF [1,2]. Our patient presented with similar clinical manifestations, with the exception of right-lobe predominant hepatomegaly. Children have a more severe and classical phenotype than adults and more severe complications of portal hypertension [11].

CD was first described in 1958 by Jacques Caroli, a French gastroenterologist [12,13]. It is classified in Todani’s group V of biliary tract diseases and defined as multifocal non-obstructive dilation of large intrahepatic biliary ducts [12,13]. It is a rare disorder with an incidence of 1:1,000,000 population and an autosomal recessive inheritance pattern [14]. When the disease is accompanied by CHF, it is called Caroli's syndrome or Grumbach’s disease [12,14]. The incidence of CS is greater than the pure form of CD [9]. Renal involvement has been described in up to 60% of cases due to mutations in the polycystic kidney and hepatic disease 1 gene (PKH1D) localized on chromosome 6. This gene codes for fibrocystin/polycystin, a protein that is expressed in the renal and biliary epithelium and maintains the three-dimensional tubular architecture [1,12,15,16]. Nevertheless, a study assessing the relationship between CHF and ARPKD concluded that the severity of the kidney disease and liver disease are independent of each other [17].

Patients with CD are often diagnosed around the age of 20, presenting with episodes of cholangitis, portal hypertension, or hepatomegaly [14]. A study demonstrated that CS is the predominant phenotype in children with an early-onset presentation. In addition, children with CS had a greater incidence of cholangitis than the ones with isolated CHF, and have a worse prognosis due to the likelihood of developing end-stage renal disease [10]. It is important to note that our patient had no history of episodes of abdominal pain, jaundice, and fever, and this was the first time he presented with upper gastrointestinal bleeding. 

Liver biopsy is essential for the definitive diagnosis of CHF [2,5]. Liver tissue shows fibrous septa periportally and interportally, causing false distortion of the lobular architecture with the creation of different size nodules, thus mimicking cirrhosis. However, there will be an absence of porto-central vein bridging and regenerative nodules [2]. Ultrasound (US) shows very bright areas of echogenicity in the liver due to the dense bands of fibrous tissue [5]. For CD, US and contrast-enhanced CT scan are employed as the first-line diagnostic modalities [3]. US reveals multiple anechoic intrahepatic bile lakes surrounding portal vein radicals [1]. Our patient’s abdominal ultrasound was suggestive of CHF, showing alterations in echogenicity. Moreover, CT angiography revealed dilated saccular bile ducts, a key characteristic of CS. However, it also revealed dilation of the common bile duct, an association not commonly described in CS. The incidence of this association is unknown, but in a study by Levy et al. involving 17 patients, extrahepatic duct dilation was found in 53% of them [18,19].

Starting at the time of initial diagnosis, all patients with ARPKD should be evaluated by a gastroenterologist and undergo abdominal US evaluations annually to detect liver abnormalities due to the high association with CS [17].

CD/CS complications include cholestasis, choledocholithiasis, and cholangitis. It also increases the risk for malignancy (seen in 7% of patients) [1,9]. None of these complications were found in our patient. As for CHF, both hepatocellular and cholangiocarcinoma may be a complication [5].

The treatment for CHF is directed toward supportive treatment and mitigating complications such as portal hypertension. Patients with variceal hemorrhage should be treated by sclerotherapy or banding. If these fail, they should undergo portosystemic shunting and splenectomy. Eventually, these patients will need a liver and/or renal transplant [5,11]. There are no guidelines on when a dual liver and kidney transplantation for ARPKD/CHF is needed [2]. For the diffuse form of CD or CS associated with fibrosis and end-stage liver disease, a liver transplant is the preferred treatment [12]. A study found the following indications for pediatric liver transplant in patients with CD/CS: recurrent cholangitis, biliary cirrhosis, and refractory upper gastrointestinal bleeding [20]. Likewise, Millwalla et al. studied 104 patients with CD/CS who underwent liver transplant and found excellent patient and graft survival. Another study reported that patients with CS presenting in childhood have a worse prognosis, and are likely to develop end-stage renal disease (ESRD) and need a combined liver-kidney transplant [10].

## Conclusions

We report a unique case of CS in a patient with past medical history of ARPKD who presented with an episode of variceal bleeding secondary to portal hypertension at two years of age. Even though the occurrence of CS is rare and its presentation in early childhood is not common, the association between ARPDK and FLD is well documented, therefore CS should be suspected early on. A close follow-up of the patient’s kidney and liver functions is key in determining treatment and need of a combined liver-kidney transplant in patients like ours with an early presentation.
